# The effects of an 8-week dynamic neuromuscular stabilization exercise on pain, functional disability, and quality of life in individuals with non-specific chronic low back pain: a randomized clinical trial with a two-month follow-up study

**DOI:** 10.1186/s13102-024-00948-9

**Published:** 2024-07-25

**Authors:** Alireza Rabieezadeh, Reza Mahdavinejad, Morteza Sedehi, Meisam Adimi

**Affiliations:** 1 Department of Sport Injuries and Corrective Exercises, Faculty of Sport Sciences, University of Isfahan, Isfahan, Iran; 2 Department of Epidemiology and Biostatistics, School of Health, Modeling in Health Research Center, Shahrekord University of Medical Sciences, Shahrekord, Iran; 3 Department of Neurosurgery, School of Medicine, Neurosciences Research Center, Kashani Hospital, Isfahan University of Medical Sciences, Isfahan, Iran

**Keywords:** Dynamic neuromuscular stabilization, Low back pain, Quality of life, Functional disability, Exercise

## Abstract

**Background:**

Low back pain (LBP) is a common disorder and is considered one of the leading causes of disability worldwide, resulting in adverse health, social, and economic outcomes. This study aimed to investigate the effects and durability of 8-week Dynamic Neuromuscular Stabilization (DNS) exercises on pain, functional disability, and quality of life in individuals aged 30 to 50 years with non-specific chronic low back pain (NSCLBP).

**Methods:**

This research employed a pre- and post-test design with a follow-up period, in which 29 participants (16 in the control group and 13 in the exercise group) remained until the end of the study. Pain intensity, functional disability, and quality of life were assessed using the visual analog scale (VAS), the Oswestry Disability Index, and the SF-36 questionnaire, respectively, before intervention, immediately after, and two months post-intervention. The control group continued their routine daily activities, while the exercise group performed DNS exercises three times a week for 8 weeks. The data was analyzed using a mixed-design ANOVA (*P* ≤ 0.05).

**Results:**

The results showed improvements in pain (F (2,24) = 5.31, *P* = 0.01, η^2^ = 0.31), functional disability (F (2,24) = 4.17, *P* = 0.03, η^2^ = 0.26), and quality of life (F (2,24) = 4.70, *P* = 0.02, η^2^ = 0.28) in the exercise group at the Post-test compared to the Pre-test. However, the durability of the exercise effects at the follow-up assessment was not sustainable compared to the Post-test and Pre-test (*P* > 0.05).

**Conclusion:**

An 8-week period of DNS exercises can improve pain, functional disability, and quality of life in individuals with NSCLBP. However, a 2-month period of detraining can reduce the positive outcomes of these exercises.

**Trial registration:**

The researchers retrospectively registered this trial on 21/04/2024, with the identifier IRCT20240107060646N1 in the Iranian Registry of Clinical Trials (IRCT) at the following address: https://irct.behdasht.gov.ir.

**Supplementary Information:**

The online version contains supplementary material available at 10.1186/s13102-024-00948-9.

## Background

Low back pain (LBP) is a common disorder, and it has been reported as a significant health, social, and economic problem that is associated with work absenteeism, disability, and high costs for both patients and society [1]. LBP has been recognized as a primary cause of disability, and 50% of individuals diagnosed with LBP in 1990 still experience disability [2]. In 85 to 90% of cases, the exact cause of pain cannot be definitively determined; therefore, patients are classified as having non-specific low back pain (NSLBP) [3]. If the pain persists for more than three months without any specific cause, such as trauma or malignancy, it is recognized as non-specific chronic low back pain (NSCLBP) [4]. Extensive research has been conducted to examine the causes and factors associated with LBP. LBP not only causes functional limitations but also adversely affects individuals’ overall health and psychological well-being, ultimately leading to a lower quality of life [5]. On the other hand, psychological factors such as fear-avoidance beliefs, depression, anxiety, family and social stress, and high levels of disability are associated with chronic low back pain (CLBP) [6]. There is also evidence of a link between LBP and urinary incontinence, respiratory problems, and gastrointestinal symptoms, where the presence of one symptom is accompanied by the occurrence of other symptoms [7]. Therefore, the association between LBP and various dimensions of life and health highlights the importance of addressing it more vigorously.

Various treatments are prescribed for LBP. However, the reported effects are modest and moderate, possibly due to a lack of precise understanding of the pathophysiology and heterogeneity of the study population [8]. Exercise is generally considered a relatively effective treatment for CLBP [1]. Zhu et al. conducted a study comparing the effects of yoga, non-exercise activities (such as usual care and education), and therapeutic exercises on pain, functional disability, and quality of life in patients with CLBP. They reported that yoga had a positive impact on reducing pain and disability in individuals with LBP. However, it may not improve their long-term physical and mental dimensions of quality of life [9]. In line with this, Yu et al. conducted another study investigating the effects of Pilates on pain, functional disability, and quality of life in patients with CLBP. They reported that Pilates may be beneficial in relieving pain and improving functional impairments in individuals with CLBP. However, the impact on improving quality of life may be less pronounced [10]. In another study aiming to investigate the short-term and midterm effects of pain neuroscience education (PNE) combined with manual therapy (MT) and home exercise program (HEP) on pain intensity, lumbar functional performance, disability, and movement fear in patients with CLBP, a multidimensional treatment program combining PNE, MT, and HEP was found to be more effective in improving lumbar function and reducing pain, disability, and movement fear in the short-term (4 weeks) and midterm (12 weeks) [11].

Therefore, the positive effects of exercises such as yoga and Pilates on CLBP may be attributed to their multidimensional intervention approach. In addition to the physical aspects, these exercises also focus on the mental dimension. By incorporating various body positions and breathing techniques, these interventions contribute to the establishment of physical and emotional balance [12].

Frank [13] stated that simply strengthening the abdominal muscles, erector spinal muscles, gluteus, or any other muscle alone cannot ensure core stability. Instead, it is achieved through precise coordination of these muscles along with the integrated stabilizing system of the spine (ISSS) and the regulation of intra-abdominal pressure (IAP) performed by the central nervous system (CNS). He reported that there are genetically encoded fundamental movement patterns present in a healthy neonate that, as the central nervous system matures, automatically activate and enable the neonate to acquire basic locomotor skills [13]. Furthermore, in a healthy neonate, muscle activation timing, balance, and interaction of muscles around joints, as well as the activation of fundamental movement patterns, all contribute to joint centration and optimize its mechanical advantage. So, if any of these systems fail to function correctly, it can lead to altered movement patterns and excessive load on the joints, resulting in functional deficits and disrupted kinetic chains [14].

It should be noted that the diaphragm initially functions as a respiratory muscle during the neonatal period. As the CNS matures and the infant assumes upright posture, typically around six months of age, coordinating abdominal and chest breathing, the diaphragm begins to perform dual functions as a respiratory and postural muscle. According to Kolar, the function of the IAP and the Integrated ISSS can be compromised due to the insufficient functioning of the postural role of the diaphragm [15]. Consequently, this inefficiency in diaphragmatic function often leads to increased compressive forces on the spine due to the compensatory activity of the superficial extensor muscles of the spine [15]. Therefore, through exercises known as dynamic neuromuscular stabilization (DNS), by placing individuals in positions resembling the first year of life and strengthening the function of the diaphragm through proper breathing exercises and recalling natural movement patterns stored in the central nervous system, it is possible to reconstruct faulty movement patterns in adulthood. In a general sense, the perspective and goal of DNS are to restore the ISSS, regulate IAP, and prevent excessive load on joints for optimal efficiency of the musculoskeletal system [13, 16].

Regardless of psychological issues, considering that one of the main problems in these patients is a deficiency in movement patterns and disruption of their natural kinetic chain, based on this theory, the effects of DNS exercises may be more effective in alleviating symptoms and problems in individuals with NSCLBP. Furthermore, the durability of the effects of DNS exercises in patients with NSCLBP has not been investigated thus far. Hence, this study aims to investigate the effects of 8 weeks of DNS exercises on pain intensity, functional disability index, and quality of life in individuals aged 30 to 50 with NSCLBP, as well as whether any changes occur in the measured indices after two months of detraining following the exercises.

## Methods

### Research design

**Fig. 1:**
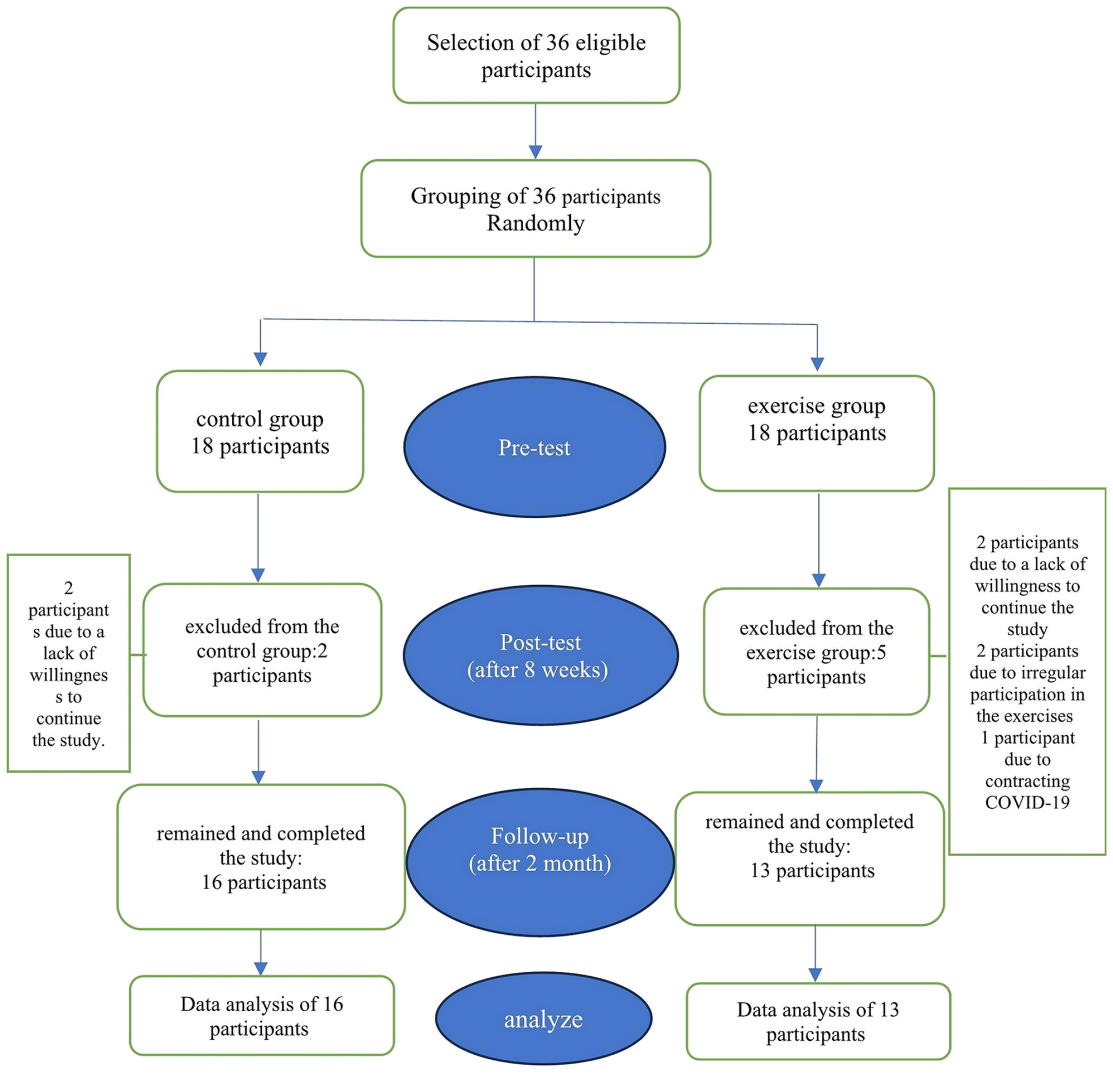
Flow chart of the study participants

The present study employed a randomized controlled trial Pre-test and Post-test with a follow-up design. One of the researchers took measurements of pain, functional disability, and quality of life in the afternoon before the exercises started, the day after the last training session, and two months later. These measurements were taken at the sports club designated for the weekly in-person training sessions. This study followed the CONSORT 2010 statement [17]. For additional information regarding the protocol for this trial, please visit https://irct.behdasht.gov.ir and refer to the IRCT20240107060646N1 reference number. The flow chart of the research design and participant grouping is depicted in Fig. 1.

### Participants

The participants of this study were individuals with LBP within the age range of 30 to 50 years in Shahrekord, Iran. They were selected to participate in the study after visiting the specialized medical clinic, and they were checked by a neurologist, who diagnosed them with non-specific CLBP. The inclusion criteria for participation in this study were: having a minimum of three months of non-specific CLBP, the ability to sit, stand, and walk without using assistive devices, and not engaging in regular exercise. The exclusion criteria included having any specific pathology such as disc protrusion, spinal injury or surgery history, neurological disorders, tumors, osteoarthritis, and rheumatoid arthritis diagnosed by a specialist physician, being diagnosed with any medical condition that restricts physical activity by a physician, experiencing unbearable pain and inability to perform exercises, potential lung tissue damage in case of COVID-19 infection, absenteeism of more than one-third of exercise sessions irregularly or absenteeism of three consecutive sessions, and a lack of willingness to continue participation in the study.

### Randomized grouping

In the present study, the participants were selected in a purposeful manner by a specialist physician, and then they were randomly assigned into one of two groups: the exercise group and the control group (18 participants for each group). The randomization of participants was carried out as follows: The names of the participants were entered in the first column of an Excel spreadsheet. Using the online website https://www.random.org, random numbers between 1 and 36 were generated, and these numbers were entered in the second column of the Excel sheet, corresponding to the participant names. The numbers in the second column were then sorted in ascending order. Consequently, the participants with numbers 1 through 18 in the second column were designated as the control group, and those with numbers 19 through 36 were assigned to the exercise group.

### Data collection tools

For pain assessment, the visual analog scale (VAS) was used. Also, the oswestry disability index questionnaire and the SF-36 questionnaire were used for functional disability assessment and quality of life, respectively.

### VAS

The measurement tool used to assess pain intensity was the VAS, a reliable and valid instrument. The measurement method involves a straight, 100-millimeter horizontal line drawn on paper. The left end of the line is labelled “No Pain,” and the right end is labelled “Worst Imaginable Pain.” Participants mark their perceived level of pain typically experienced by placing a dot on this line, and the trainer measures the distance from the beginning of the line using a ruler and records the individual’s pain level [18]. The following cut-off points on the VAS have been recommended: no pain (0–4 millimeters), mild pain (5–44 millimeters), moderate pain (45–74 millimeters), and severe pain (75–100 millimeters) [19].

### Oswestry disability index questionnaire

Functional disability was measured using the oswestry disability index questionnaire, which has been reported as a reliable and valid tool for assessing functional limitations, particularly in individuals with LBP [20]. This questionnaire comprises ten sections: pain intensity, personal tasks, lifting, walking, sitting, standing, sleeping, sexual activity, social life, and traveling. Each section measures the level of disability on a scale of 0 to 5 (ranging from no disability to severe disability). The total score of this questionnaire is multiplied by 2 to obtain a score ranging from 0 to 100. Scores from 0 to 20 indicate a low disability, scores from 21 to 40 indicate a moderate disability, scores from 41 to 60 indicate a high disability, scores from 61 to 80 indicate a severe disability. and scores above 80 indicate a complete or near-complete disability in performing daily activities [20].

### SF-36 questionnaire

The SF-36 questionnaire, also known as the 36-item short form health Survey, is an internationally standardized tool to measure health status and satisfaction with one’s current condition. It consists of 36 questions and is composed of eight subscales, with each subscale containing 2 to 10 questions. The eight subscales of this questionnaire are as follows: physical functioning, role limitations due to physical health, bodily pain, general health perceptions, vitality, social functioning, role limitations due to emotional problems, and mental health. Additionally, from the combination of these subscales, two overall subscales, physical health and mental health, are derived, and the overall score of quality of life is calculated from the sum of these two general subscales. In this questionnaire, lower scores indicate a lower quality of life, while higher scores indicate a higher quality of life [21].

### Exercise protocol

Individuals in the control group were asked to continue their daily routine activities, while individuals in the exercise group were invited to participate in an exercise program. The exercise group engaged in stability core exercises based on the DNS approach, including Diaphragmatic Breathing, Baby Rock (supine 90–90^1^), Prone, Rolling, Side Lying, Oblique Sit, Tripod, Kneeling, Squat, and Czech Get Up [13, 14] for 8 weeks, with 3 sessions per week (see Appendix). One session was conducted in-person, while the other two sessions were conducted non-in-person (at home) due to the limitations of gatherings as a result of the COVID-19 pandemic. The in-person training sessions were held every Thursday at 4 PM at a designated sports club. To provide a flexible schedule, home training sessions were coordinated with the exercise group between 4 PM and 6 PM on Saturdays and Mondays, for a duration of 50 to 60 min.

In the first session, the trainer demonstrated the correct execution of each position and provided relevant explanations. Simultaneously, with the trainer’s assistance, the movements were recorded as videos from different angles. Afterward, all participants performed the exercise protocol, and the trainer supervised their proper execution. At the end of the session, coordination was made with the participants to conduct two remote exercise sessions (on Saturdays and Mondays) each week. A WhatsApp group was created to share the videos of exercises. It was explained that before each at-home session, participants should watch the recorded video of each exercise to refresh their memory and pay attention to the provided instructions. In the subsequent sessions, at the beginning of each in-person session (every Thursday), participants performed the same standardized warm-up as it is known that different types of warm-ups can affect exercise performance [22]. The warm-up consisted of aerobic running and static stretching exercises. Then, considering joint centration and proper breathing, new exercises were introduced based on the participants’ abilities and progress. Additionally, on the non-in-person days, the trainer maintained contact with the participants in the exercise group through phone calls to remind them of their exercise performance. It was also arranged that, in case of any ambiguity or questions arising during the exercises, the trainer would be prepared to go online, providing visual supervision and addressing inquiries through video communication.

### Statistical analysis

The sample size for repeated measures analysis of variance (ANOVA) was calculated using an effect size of 0.25, a significance level of 0.05, and a statistical power of 0.85 using G*Power software (version 3.9.1.6), a total of 32 participants (16 participants in each group) were determined. Considering the circumstances of the COVID-19 pandemic and the anticipated possibility of dropouts in the groups, 18 participants were allocated to each group. The collected data were analyzed using SPSS version 27. After checking the normality distribution with the Shapiro-Wilk test, descriptive statistics were used to examine the mean and standard deviation of the demographic and participant characteristics. The analysis of variance (ANOVA) method with mixed design (2 × 3) was employed to investigate group-related trends, and Student’s t-tests were used to examine intergroup differences at a significance level of 0.05.

## Results

A total of 29 participants, consisting of 19 females and 10 males, aged between 30 and 50 years (16 in the control group and 13 in the exercise group), completed the study. Their characteristics are presented in Table 1. This table also shows that the differences in characteristics of both groups are not statistically significant (*P* > 0.05). Additionally, the results of the RM-ANOVA statistical tests are reported in Table 2 (Table 3).

**Table 1:** Demographic and participant characteristics in the pre-test (Mean ± Standard deviation)

Variable	Control groupe (*n* = 16)	Exercise groupe (*n* = 13)	*P* (sig)
Age (year)	39.13 ± 5.52	41.77 ± 7.15	0.27
Height (cm)	166.19 ± 8.14	166.00 ± 8.33	0.95
Weight (kg)	68.91 ± 9.64	71.90 ± 8.87	0.40
BMI (kg/m^2^)	24.87 ± 2.59	26.16 ± 3.24	0.25
Pain	39.88 ± 20.83	39.31 ± 17.09	0.94
Functional disability	14.13 ± 7.95	18.31 ± 7.02	0.15
Quality of life	67.67 ± 17.19	66.14 ± 16.19	0.81

*cm* centimeter, *kg* kilogram, *m* meter, *BMI* Body Mass Index, *sig* significant difference between groups (*P* ˃ 0.05)

**Table 2:** Results of within-between group mixed-design analysis of variance (ANOVA)

Variable	Main effect							Interaction effect		
	Time			group				Group × Time		
	F	***P***	Eta-squared (η^2^)		F	***P***	Eta-squared (η^2^)	F	***P***	Eta-squared (η^2^)
Pain	2.01	0.14	0.07		4.21	0.05^*^	0.13	3.23	0.05^*^	0.11
Functional disability	3.13	0.05^*^	0.10		0.01	0.93	0.00	3.55	0.04^*^	0.12
Quality of life	3.18	0.05^*^	0.11		0.54	0.47	0.02	2.32	0.11	0.08

^*^ = *P* ≤ 0.05

**Table 3:** Student’s t-tests for comparing pain, functional disability, and quality of life between the two groups

Variable	Measuring tool	Group	Pre-test^1^			Post-test^2^			Flow-up^3^		
			Mean Difference	***F***	***P***	Mean Difference	***F***	***P***	Mean Difference	***F***	*P*
Pain	VAS (0–100 mm)	Control	0.57	0.01	0.94	21.88	11.94	0.00^*^	12.53	0.19	0.16
		Exercise									
Functional disability	Oswestry Index (0-100 point)	Control	4.18	2.20	0.15	2.50	0.63	0.43	0.91	0.07	0.79
		Exercise									
Quality of life	SF-36 Questionnaire (0-100 point)	Control	1.53	0.06	0.81	7.62	1.70	0.20	5.78	1.00	0.33
		Exercise									

*VAS* Visual Analogue Scale, *mm* millimeter, *SF-36* Short Form 36 Health Survey Questionnaire, ^1^Baseline (session 0), ^2^post-intervention (after session 24), ^3^Two month after post-intervention, ^*^ = *P* ≤ 0.05

**Table 4:** The Bonferroni post hoc test to compare variables at measurement times

Variable	Group	Pretest - posttest		Pretest – flow-up		Posttest – flow-up	
		Mean difference	*P*	Mean difference	*P*	Mean difference	*P*
Pain	Control	3.62	1.00	6.50	1.00	2.87	1.00
	Exercise	17.69	0.00^*^	5.46	1.00	12.23	0.27
Functional disability	Control	0.06	1.00	0.94	1.00	0.87	1.00
	Exercise	6.62	0.02^*^	4.15	0.33	2.46	1.00
Quality of life	Control	1.05	1.00	0.18	1.00	1.23	1.00
	Exercise	10.19	0.01^*^	7.12	0.36	3.07	0.94

^*^ = *P* ≤ 0.05

### The effect of DNS exercises on pain

**Fig. 2:**
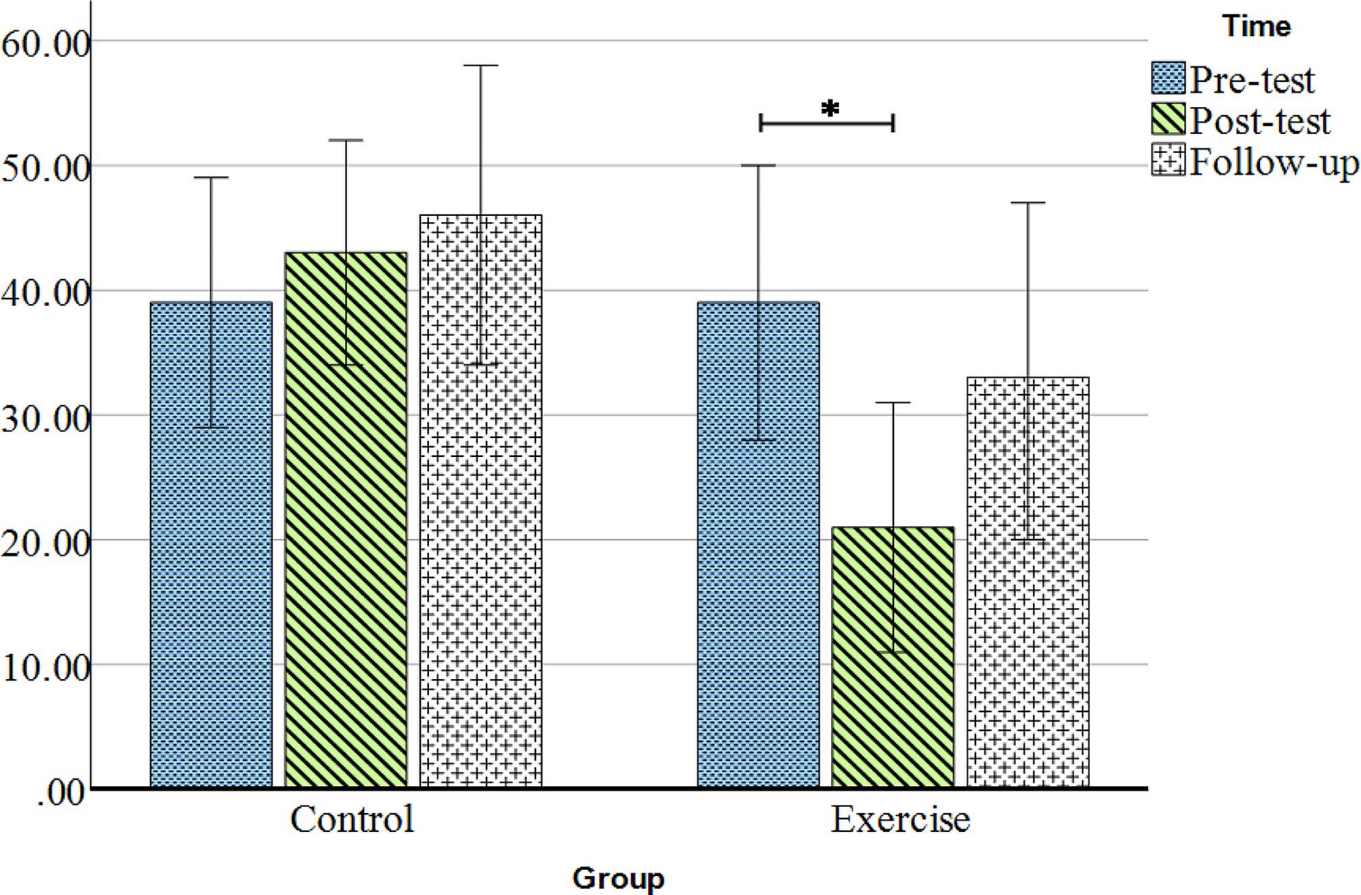
Comparison of pain changes in the two groups during the Pre-test, Post-test, and follow-up. Y-axis is pain change score, Data are presented as the mean ± standard error of the mean, ^*^ = *P* ≤ 0.05

Figure 2; Table 2 depict the changes in pain over the measurement periods. Figure 2 shows that the pain in the control group is relatively similar in the Post-test compared to the Pre-test and increases slightly during the follow-up. However, in the exercise group, pain decreases in the Post-test and increases again during the follow-up. The Mauchly’s test of sphericity indicates the similarity of variances between the two groups (X^2^ (2) = 1.85, *P* = 0.40). Mixed-design analysis of variance reveals a significant time × group interaction (F (2,54) = 3.23, *P* = 0.05, η^2^ = 0.11). Intergroup comparisons indicate a significant difference in pain between the two groups in the Post-test (F (1, 27) = 11.94, *P* < 0.01, d = 1.29) (Table 3). Also, for a detailed examination of the interaction effect, a repeated measures analysis of variance is used for each group. The results demonstrate a significant trend in pain changes in the exercise group (F (2, 24) = 5.31, *P* = 0.01, η^2^ = 0.31), while it is not significant in the control group (F (2, 30) = 0.57, *P* = 0.57, η^2^ = 0.04). Furthermore, the Bonferroni post hoc test reveals a significant difference between the Pre-test and Post-test in the exercise group (*P* < 0.001). However, there was no statistically significant difference between the Pre-test and follow-up, as well as between the Post-test and follow-up (*P* > 0.05) (Table 4).

### The effect of DNS exercises on functional disability

**Fig. 3:**
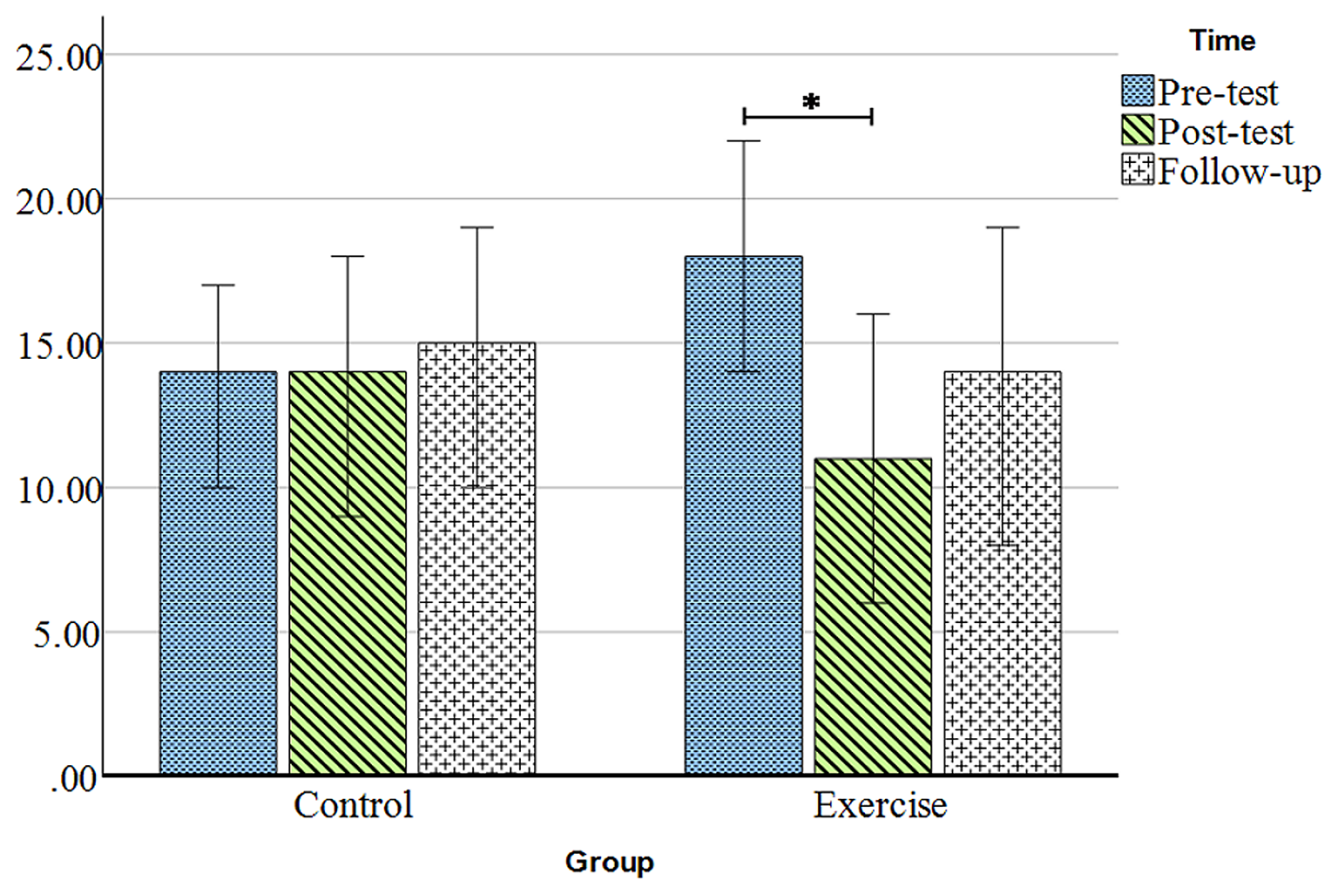
Comparison of functional disability changes in the two groups during the Pre-test, Post-test, and follow-up. Y-axis is Oswestry change score, Data are presented as the mean ± standard error of the mean, ^*^ = *P* ≥ 0.05

Figure 3; Table 2 display the changes in functional disability over the measurement periods. Figure 3 shows that the functional disability in the control group is relatively similar in the Post-test compared to the Pre-test and increases slightly during the follow-up. However, in the exercise group, functional disability decreases in the Post-test and increases again during the follow-up. The Mauchly’s test of sphericity indicates the similarity of variances between the two groups (X^2^ (2) = 2.27, *P* = 0.32). Mixed-design analysis of variance reveals a significant time × group interaction (F (2,54) = 3.55, *P* = 0.04, η^2^ = 0.12). There were no significant differences observed between the two groups in the comparison of measurement time points (*P* > 0.05) (Table 3). Therefore, for a detailed examination of the interaction effect, a repeated measures analysis of variance is used for each group. The results demonstrate a significant trend in functional disability changes in the exercise group (F (2, 24) = 4.17, *P* = 0.03, η^2^ = 0.26), while it is not significant in the control group (F (2, 30) = 0.27, *P* = 0.77, η^2^ = 0.02). Additionally, the Bonferroni post hoc test reveals a significant difference between the Pre-test and Post-test in the exercise group (*P* = 0.02). However, there was no statistically significant difference between the Pre-test and follow-up, as well as between the Post-test and follow-up (*P* > 0.05) (Table 4).

### The effect of DNS exercises on quality of life

**Fig. 4:**
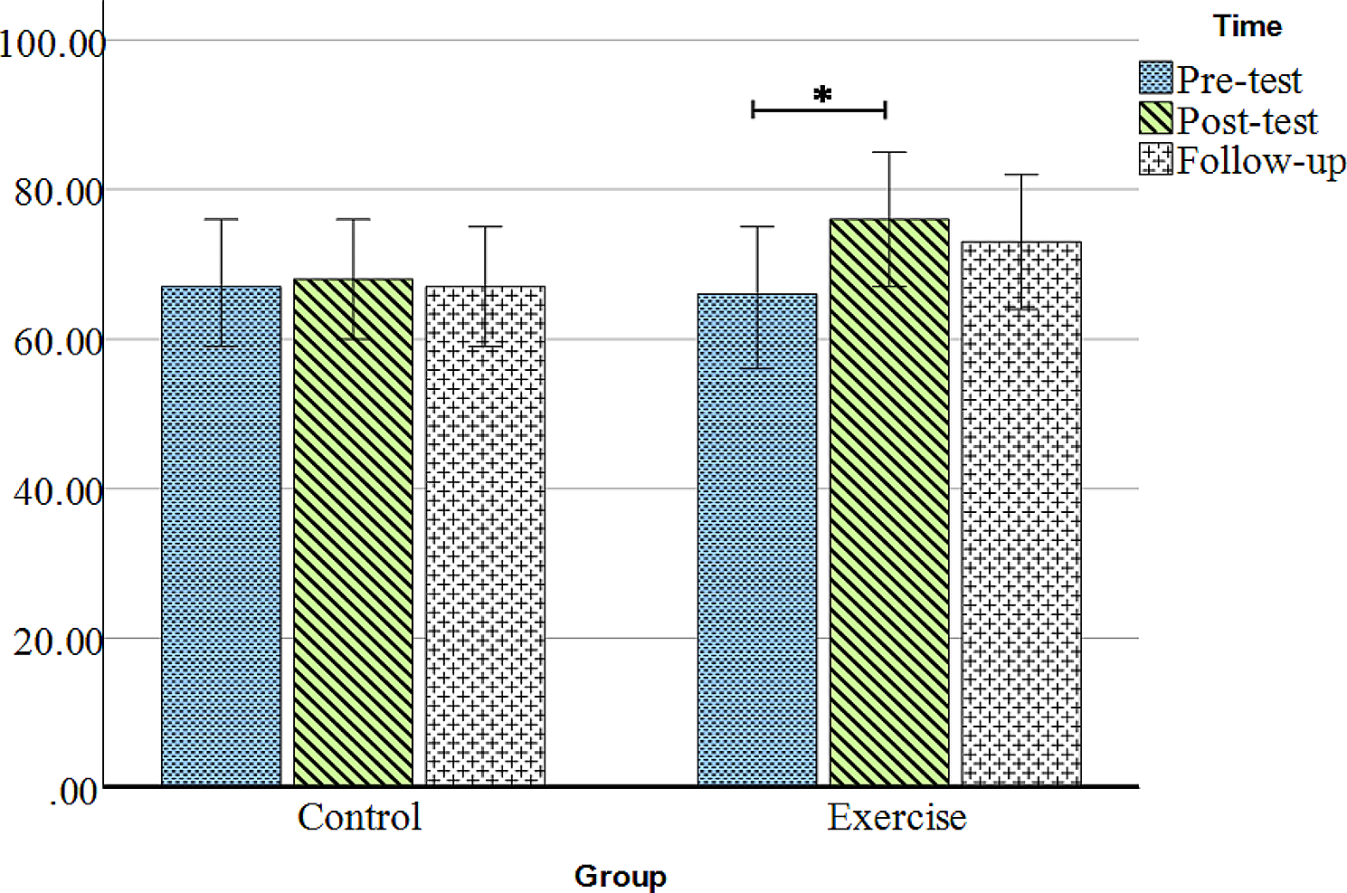
Comparison of quality of life changes in the two groups during the Pre-test, Post-test, and follow-up. Y-axis is quality of life change score, Data are presented as the mean ± standard error of the mean, ^*^ = *P* ≤ 0.05

Figure 4; Table 2 depict the changes in quality of life over the measurement periods. Figure 4 shows that the quality of life in the control group is relatively similar across the three measurement time points, while in the exercise group, the quality of life increases in the Post-test and decreases during the follow-up. The Mauchly’s test of sphericity indicates the similarity of variances between the two groups (X^2^ (2) = 4.54, *P* = 0.10). Mixed-design analysis of variance reveals a significant main effect of time points on quality of life (F (2,54) = 3.18, *P* = 0.05, η^2^ = 0.11), but the time × group interaction effect is not significant (F (2,54) = 2.32, *P* = 0.11, η^2^ = 0.08). Therefore, a repeated measures analysis of variance is used for each group to examine the main effect more closely. The results demonstrate a significant trend in quality of life changes in the exercise group (F (2, 24) = 4.70, *P* = 0.02, η^2^ = 0.28), while it is not significant in the control group (F (2, 30) = 0.10, *P* = 0.90, η2 = 0.01). The Bonferroni post hoc test reveals a significant difference between the Pre-test and Post-test in the exercise group (*P* = 0.01). However, there was no statistically significant difference between the Pre-test and follow-up, as well as between the Post-test and follow-up (*P* > 0.05) (Table 4).

## Discussion

This study aimed to investigate the effect and durability of an 8-week DNS exercise program on pain, functional disability, and quality of life in individuals aged 30 to 50 with NSCLBP. The results demonstrated that these exercises led to a reduction in pain and functional disability and an improvement in the participants’ quality of life in the Post-test compared to the Pre-test. Additionally, for the first time, the long-term effect of these exercises on CLBP was evaluated. The effect of this type of exercise decreased after a period of detraining, but it did not return to the initial baseline level.

Reviewing previous research on the effect of DNS exercises on individuals with non-specific CLBP reveals that few quantitative studies have been conducted in this area, possibly due to the novelty of this type of exercise compared to conventional exercises. Additionally, existing studies have reported the positive effects of DNS exercises on reducing pain and disability in the studied groups [23–25]. For example, Ghavipanje et al. investigated the effects of 6 weeks of DNS exercises on postpartum LBP in obese women. They reported that the participants’ pain and disability decreased due to these exercises [23]. Similarly, Najafi Ghagholestani et al. conducted a comparison between DNS exercises and conventional water exercises for individuals aged 30 to 50 years with NSCLBP. They observed that both types of exercises had positive and similar effects on reducing pain and disability, suggesting that DNS exercises could serve as an alternative to water exercises for these individuals [24]. Furthermore, even in a study that examined the effects of DNS exercises on individuals with intervertebral disc degeneration, it was reported that after 8 weeks of DNS exercises, regardless of the patient’s age, their pain and disability decreased [25].

The probable mechanism of influence in this type of exercise may be due to the target muscles and their activation during these exercises. According to the DNS approach, in the ISSS provided by Frank, there is a muscular balance between the deep neck flexors and the spine extensors in the cervical and upper thoracic regions, as well as the diaphragm, pelvic floor, all abdominal muscles, and the spine extensors in the lower thoracic and lumbar regions. Additionally, the diaphragm, pelvic floor, and transverse abdominal muscles regulate IAP and provide stability in the anterior pelvic-lumbar area [13]. It is evident that most of these muscles form the core. The role of core muscles in improving LBP and functional disability has been discussed in various studies [26, 27].

There has been little scientific research on the impact of DNS exercises on participants’ quality of life. In this regard, Marinkovic et al. investigated the effect of DNS exercises on the quality of life of healthy individuals after COVID-19. They found that an 8-week exercise program led to an improvement in the exercise group’s quality of life compared to the control group [28]. Another study examined the impact of DNS exercises on pain, disability, and quality of life in patients following shoulder arthroscopy, revealing a decrease in pain and disability and an enhancement in the individuals’ quality of life [29]. Karartı et al. also compared the effects of physiotherapy with DNS exercises in 6 weeks (3 days per week) on non-specific chronic back pain in elderly individuals. In the DNS group, they observed more remarkable improvement in the overall score of the Functional Movement Screen (FMS) and most of its items (deep squat, lunge, hurdle step, shoulder flexibility, trunk stability) compared to the physiotherapy group. However, in terms of quality of life, the improvement between the two groups was positive and similar [30]. Therefore, considering that DNS exercises target the core muscles more and FMS tests also assess the core muscles to a greater extent, it was predicted that these exercises would likely result in increased FMS scores [31]. Therefore, the present study’s findings align with the outcomes of earlier research in this field. Other investigations have also shown that therapeutic exercises lead to improvements in mobility, functional disability, and quality of life in individuals with CLBP [32].

In terms of the exercise effect’s durability, the results of this study indicated that, although the levels of pain and disability increased and quality of life decreased after two months of detraining following the completion of the exercises, they did not return to the baseline level of the initial assessment. The review of the studies conducted using the DNS exercise method on patients with LBP did not yield a research paper investigating the durability of DNS exercises. Coulombe et al. compared the effects of core stability exercises and general exercises on back pain in individuals. They observed that during the 3-month exercise period, core stability exercises were more effective than general exercises. However, the 6-month and 12-month follow-up studies revealed no significant difference in the durability of exercise effects between the two groups. [33]. Similarly, another study investigated the effects of Pilates exercises on the disability of patients with LBP and observed that this type of exercise improved the patients’ performance. However, this improvement decreased after 13 weeks of detraining [34]. In another study that aimed to determine the short-term and long-term effectiveness of using Pilates along with physiotherapy compared to physiotherapy alone in a population for postmenopausal women with CLBP, it was observed that the use of Pilates along with physiotherapy provided better outcomes in pain management and functional status of postmenopausal women with CLBP, and its effects persisted even after one year [35]. Mehling et al., too, conducted a study comparing respiratory exercises with physiotherapy for CLBP. They observed that respiratory exercises can have a positive effect similar to high-quality physiotherapy on pain, disability, and quality of life in the short term (6 to 8 weeks) and long term (6 months). However, the short-term effect favors respiratory therapy, while the long-term effect favors physiotherapy [36].

It is important to consider that the mere designation of an exercise modality is not a sufficient criterion for determining its effectiveness. In a recent review, Patti et al. examined the effect of Pilates exercise on individuals with chronic low back pain. The results show that Pilates exercise is better than no exercise or minimal exercise interventions at managing pain and disability [37, 38]; however, it is hard to pinpoint the exact factors or types of Pilates exercises that led to these improvements because the study groups were different and the exercise programs varied in terms of intensity, duration, and variety [37]. Therefore, to facilitate a more accurate comparison of the effectiveness of different exercise modalities, it is preferable, whenever possible, to utilize homogeneous study groups with comparable exercise program characteristics.

It should be noted that the reason researchers conducted this study on the effects of DNS exercises on individuals with LBP was not only due to the novelty of the exercise type and the examination of the durability of the effects but also because the study was conducted during the COVID-19 pandemic. Part of the exercises were performed in the presence of a trainer, while another part was done at home without a trainer. Nevertheless, recent research has reported the negative impacts of COVID-19 on the quality of life of individuals [39]. Researchers have also reported that the COVID-19 pandemic has significantly impacted pain levels, mental health, and quality of life in patients with conditions like LBP and chronic musculoskeletal pain, resulting in worsened pain, mental health, and quality of life [40]. It appears that the social restrictions imposed during the COVID-19 pandemic, such as staying at home, travel limitations, and particularly reduced physical activity [41], as well as limited access to non-invasive treatments like physical activity, have had an impact on individuals’ quality of life during this period [42]. However, in this study, the exercise group showed improvements in pain, functional disability, and quality of life after the intervention. Perhaps if the pandemic conditions were not present, the results would have demonstrated even more significant improvements.

It is important to note that the DNS approach focuses on the subcortical level, aiming to provide functional stability by emphasizing core body stability and recalling natural movement patterns stored in the CNS [43]. In this regard, a study evaluated the effects of exercise on brain function, pain perception, and quality of life in adults with chronic pain, including LBP, and found that exercise interventions lasting 12 weeks or more altered brain function while improving pain and quality of life. Therefore, through proper planning (i.e., intervention duration), exercise may be a suitable option for managing chronic pain by positively impacting brain health [44]. Thus, considering the DNS perspective and the findings of Palmer’s study, it is likely that better results would have been obtained if the duration of the exercise intervention in the current study had been longer.

According to research that compares exercise methods with physiotherapy [35, 45], the use of specific exercise methods, particularly DNS exercises, may have an advantage over physiotherapy. This is because DNS exercises not only strengthen and rehabilitate muscles but also invoke healthy movement patterns from the central nervous system through exercises within the range of motion, leading to the reconstruction of faulty movement patterns. This advantage, in turn, may contribute to the long-term sustainability of exercise effects compared to non-exercise methods. In this regard, Lee et al. compared DNS exercises with conventional core stability exercises in stroke patients. They reported that DNS exercises not only showed better results in the timing of controlling postural muscles, but also had a more significant long-term effect on reducing the fear of falling in these individuals compared to conventional core stability exercises [46]. Therefore, the duration of intervention, type of intervention, integration of therapeutic approaches, and duration of detraining until follow-up assessment appear to be factors influencing the durability of exercise effects, but overall evidence suggests that the positive effects of the intervention diminish after a period of detraining.

In the present study, we used the VAS, Oswestry Disability Index questionnaire, and SF-36 questionnaire due to their accessibility and ease of assessment. Scientific research has reported the validity of these questionnaires [18, 20, 21], but their use poses a potential limitation. Knowing they are in the study, participants may unintentionally respond in a way they think will please the researchers (reference). [47]. This bias was one of the limitations of the present research. Also, one of the reasons for using both in-person and non-in-person exercises was to encourage participant cooperation in the research project under the conditions of the COVID-19 pandemic and minimize gatherings in exercise sessions. During the in-person sessions, the researcher tried to remind the participants of possible points and errors they might encounter when practicing at home, but it cannot be confidently affirmed that the exercises were executed correctly without the supervision of a trainer at home. Indeed, this issue was another of the study’s limitations.

Based on the results of the present research and previous studies, it appears that DNS exercises have the potential to influence the CNS and assist in the rehabilitation of dysfunctional movement patterns. Furthermore, the integration of respiratory exercises with motor control exercises in this training method may have influenced its effectiveness. Therefore, educating patients and healthcare providers about the benefits of DNS exercises and the importance of regular physical activity for preventing NSCLBP could aid in treatment management. In this regard, it is recommended to conduct a study comparing the effects and durability of DNS exercises with other therapeutic exercises in this field. Such a study would provide valuable insights into the relative effectiveness and long-term benefits of DNS exercises compared to other therapeutic interventions.

## Conclusions

The findings of this study demonstrated that an 8-week DNS exercise program led to improvements in pain, functional disability, and quality of life in individuals aged 30 to 50 with NSCLBP. Furthermore, although the positive effects of the exercises moderated after a 2-month follow-up study, they did not return to the initial baseline level. Previous research has shown that core body muscle exercises and respiratory muscle exercises have positive effects on LBP. In the DNS approach, both core body muscles and respiratory muscles are exercised simultaneously, which sets it apart from other approaches and may be a beneficial aspect of DNS exercises. It is important to note that the positive effects of the exercises may decrease after a period of detraining. This emphasizes the significance of adopting regular exercise and maintaining an active lifestyle to sustain the benefits gained from exercises.

### Electronic supplementary material

Below is the link to the electronic supplementary material.

Supplementary Material 1

## Data Availability

The data used in the current study are available from the corresponding author upon reasonable request.

## Footnotes

1 . Hips and knees to 90° flexion.

## Abbreviations

LBP: Low back pain
NSLBP: Non-specific low back pain
NSCLBP: Non-specific chronic low back pain
CLBP: Chronic low back pain
PNE: Pain neuroscience education
MT: Manual therapy
HEP: Home exercise program
ISSS: Integrated stabilizing system of the spine
IAP: Intra-abdominal pressure
CNS: Central nervous system
DNS: Dynamic neuromuscular stabilization
VAS: Visual analog scale
