# Supplementary material for: The effects of an 8-week dynamic neuromuscular stabilization exercise on pain, functional disability, and quality of life in individuals with non-specific chronic low back pain: a randomized clinical trial with a two-month follow-up study
Source: BMC Sports Sci Med Rehabil. 2024 Jul 25;16:161. doi: 10.1186/s13102-024-00948-9 (PMC11271024; doi:10.1186/s13102-024-00948-9)

**Appendix**

**DNS exercise Considerations:**

1. The images illustrate the overall structure of the exercises based on growth positions. It should be noted that resistance exercises are not performed before achieving proper stability and mastery of breathing.
2. During the first week of training, exercises are performed without using weights or resistance. The focus is on holding positions for 10 seconds, with 10 repetitions and approximately 2 minutes of rest between each repetition.
3. Throughout all training stages, proper diaphragm breathing and control of intra-abdominal pressure are emphasized.
4. Progression and increasing exercise load have been planned from a more straightforward level (position B) to a more challenging and demanding level (position D).
5. By individual abilities and considering the principle of individual differences, once the patient has comfortably mastered their exercise position, progression is given by moving the limbs against resistance bands, Swiss balls, or using weights.
6. When exercising against resistance, the resistance level is adjusted based on the weakest part of the stabilization system.
7. As long as the patient maintains coordination and an ideal breathing pattern, necessary modifications and advancements can be made.
8. The number of repetitions is performed only to the extent to which the patient can demonstrate flawless stability and high-quality movement patterns.

**DNS Exercise Program**

| Week | Session | Position | Type of Exercise | Repetition/  Hold (Seconds) | Sets | Rest time (seconds) | | Description^*^ |
| --- | --- | --- | --- | --- | --- | --- | --- | --- |
| 1 | 1-3 | B | Isometric | 10 S | 1 to 3 | 90 to 120 | α | |
| 2 | 4-6 | C | Isometric | 10 S | 2 to 3 | 90 to 120 |  |  |
| 3 | 7-9 | C | Isotonic | 10 R | 2 to 3 | 90 to 120 |  |  |
| 4 | 10-12 | D | Isotonic | 10 R | 2 to 3 | 90 to 120 |  |  |
| 5 | 13-15 | D | Isotonic | 10 to 15 R | 2 to 3 | 90 to 120 |  |  |
| 6 | 16-18 | D | Isotonic | 10 to 15 R | 3 | 90 |  |  |
| 7 | 19-21 | Czech Get-Up | Isometric and functional | 1 repetition for each position | 10 | 90 to 120 | β | |
| 8 | 22-24 | Czech Get-Up | Functional | 1 repetition from position 1 to 10 | 10 | 90 to 120 | γ | |

^*^ = At the beginning of each training session, a warm-up of approximately 5 to 7 minutes is performed, and at the end of each training session, a cool-down of approximately 5 to 7 minutes is also done. Furthermore, in all exercises, the intensity of the training can vary based on the individual's ability.

α = In isometric exercises, diaphragmatic breathing is performed with the nose to inhale and the mouth to exhale at 3 sets and 15 repetitions. 1 second inhale, 2 seconds of exhale (set 1), 2 seconds of inhale: 4 seconds of exhale (set 2), 3 seconds of inhale: 6 seconds of exhale (set 3).

β = Exercise performed intermittently, where the individual performs each position (1 to 10) sequentially with a 10-second isometric hold for each position.

γ = Exercise performed consecutively: If performed correctly, with proper breathing and intra-abdominal pressure control, kettlebells or dumbbells are used as resistance.

**Progression spectrum and resistance application based on an individual's ability over an 8-week training period**

**B**

**D**


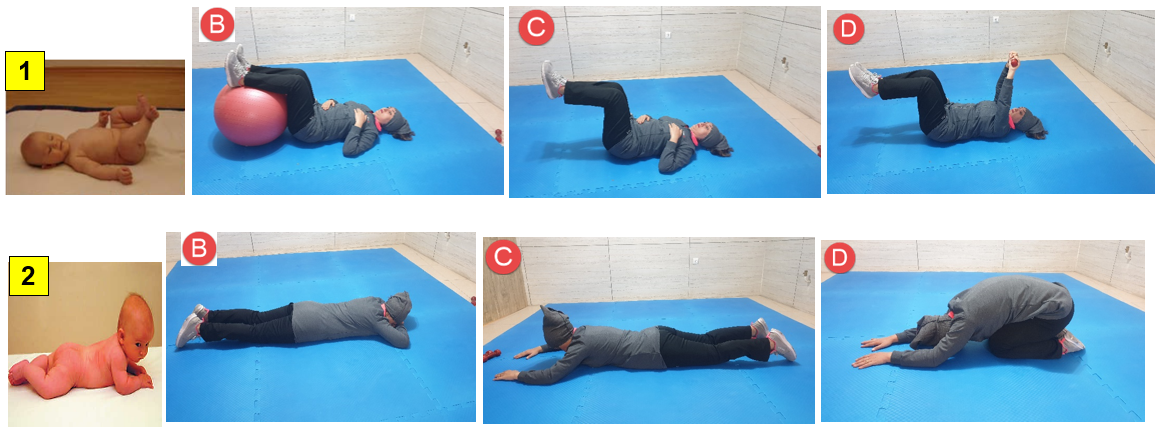


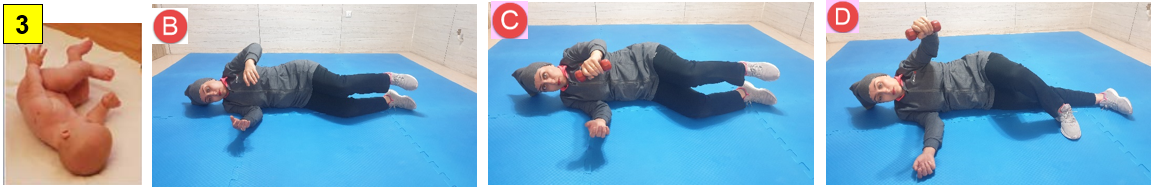


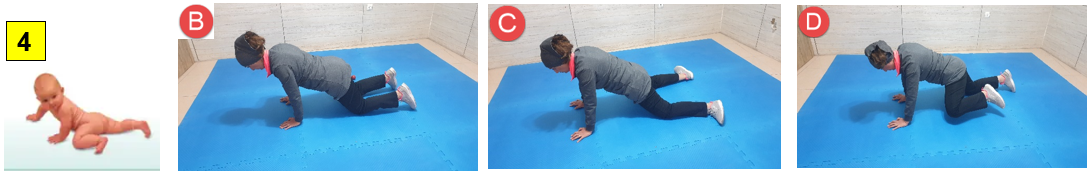


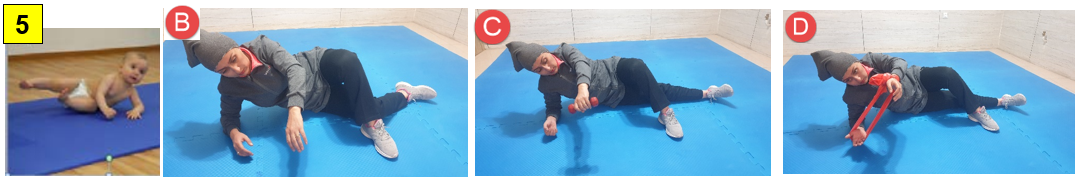


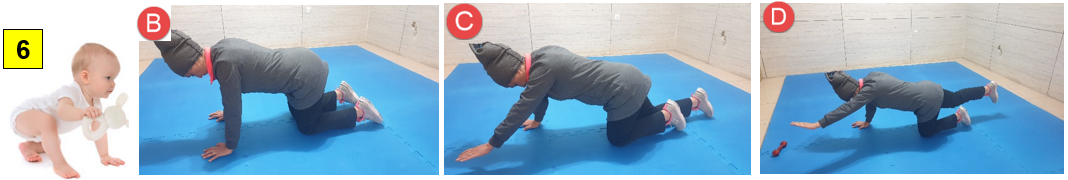


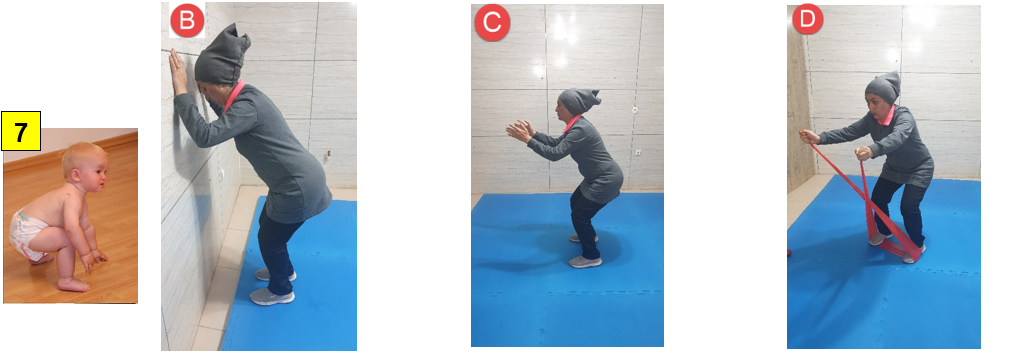


Czech Get-Up


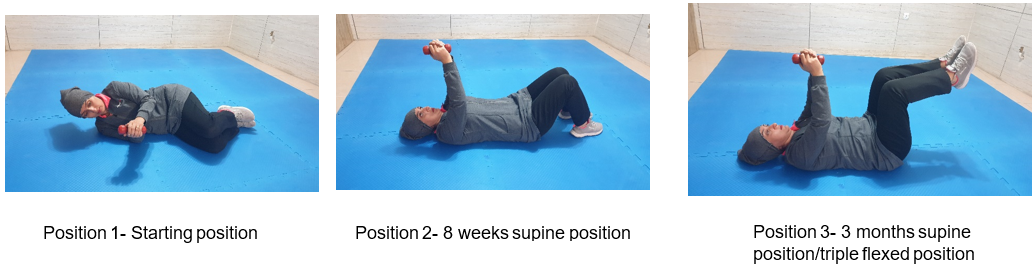


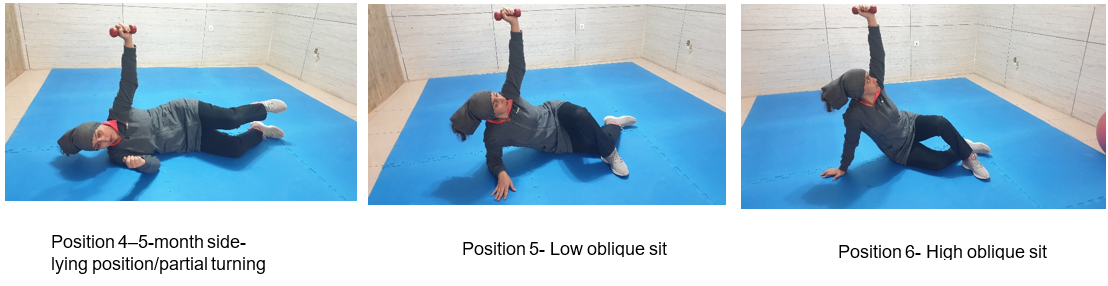


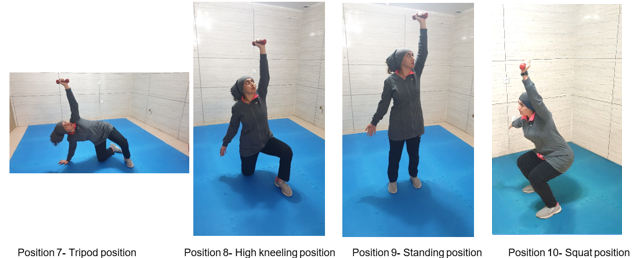

Supplement: Supplementary file 1 — Supplementary Material 1 [file 13102_2024_948_MOESM1_ESM.docx]
